# Limitations of current techniques in clinical antimicrobial resistance diagnosis: examples and future prospects

**DOI:** 10.1038/s44259-024-00033-8

**Published:** 2024-06-17

**Authors:** Jack Hassall, Carmen Coxon, Vishal C. Patel, Simon D. Goldenberg, Chrysi Sergaki

**Affiliations:** 1 Science Research and Innovation, Medicines and Healthcare products Regulatory Agency, Blanche Lane, South Mimms, Potters Bar, Hertfordshire, EN6 3QG UK; 2 The Roger Williams Institute of Hepatology London, Foundation for Liver Research, 111 Coldharbour Lane, London, SE5 9NT UK; 3 Institute of Liver Studies, School of Immunology and Microbial Sciences, Faculty of Life Sciences and Medicine, King’s College London, 125 Coldharbour Lane, London, SE5 9NU UK; 4 Institute of Liver Studies, King’s College Hospital NHS Foundation Trust, Denmark Hill, London, SE5 9RS UK; 5 Centre for Clinical Infection and Diagnostics Research, Guy’s and St Thomas’ NHS Foundation Trust and King’s College, London, UK

**Keywords:** Health services, Infectious-disease diagnostics, Antimicrobial resistance, Next-generation sequencing

## Abstract

Antimicrobial resistance is a global threat to public health. Without proactive intervention, common infections may become untreatable, restricting the types of clinical intervention that can be undertaken and reversing improvements in mortality rates. Effective antimicrobial stewardship represents one approach to restrict the spread of antimicrobial resistance but relies on rapid and accurate diagnostics that minimise the unnecessary use of antibiotics. This is increasingly a key unmet clinical need. In this paper, we describe existing techniques for the detection of antimicrobial resistance, while examining their drawbacks and limitations. We also discuss emerging diagnostic technologies in the field, and the need for standardisation to allow for swifter and more widespread clinical adoption.

## Introduction

Since the introduction of penicillin during World War II, antibiotics have become the backbone of modern medicine^1,2^. The success of antibiotics resulted in a golden age in medicine, but this is now coming to an end as we risk entering a post-antibiotic era. A lack of effective antibiotics reduces our capacity to respond to outbreaks of infectious disease. Without coordinated, proactive interventions to detect and manage antimicrobial resistance (AMR), there will be a significant regression in medical care and a steep increase in mortality rates^3^. Many modern medical techniques are dependent on the availability of effective antimicrobials, without them many common procedures and interventions (cancer chemotherapy, organ transplantation, prosthetic joint replacement etc) may not be able to be undertaken without excess risk^3^.

Multi-drug resistant organisms (MDROs) are the outcome of years of antibiotic dependency in medical practice and are responsible for an increasing number of infections. MDRO are categorised by three increasing resistance levels^4^:

- Multidrug-resistant (MDR) – nonsusceptibility to at least one agent in three or more antimicrobial agent classes.
- Extensively drug-resistant (XDR) – nonsusceptibility to at least one agent in all but two (or fewer) antimicrobial agent classes.
- Pan drug-resistant (PDR) or sometimes referred to as totally drug-resistant (TDR) whereby the organism shows nonsusceptibility to all agents in all classes.

MDROs are considered a global crisis affecting low, middle and high-income countries^5^, given their potentially untreatable nature^6^. AMR does not respect borders, neither geographical nor ecological, and with the use of antimicrobials in food producing animals we are seeing transmission of resistant pathogens from livestock into humans^7^. In 2015, the Global Action Plan on Antimicrobial Resistance was established by the World Health Assembly to address the threat of AMR^5,8^. This was followed by the United Nations General Assembly that passed a resolution unanimously calling for a globally coordinated action resulting in the One Health approach to AMR^5,9,10^. The One Health approach is a multidisciplinary joint effort to provide solutions for human, animal, and environmental health^11^.

The socioeconomic burden of AMR is difficult to gauge but known to be significant. Mortality estimates range from 0.7–4.95 million deaths worldwide annually, and healthcare costs amounting to tens of billions of US dollars^8,10,12–14^. It is likely that these numbers are an underestimate due to insufficient national reporting rates, a lack of comprehensive data coverage and no International Classification of Diseases (ICD-10) code specifically for MDRO infections^15^. In 2018, Burnham et al. re-analysed the 2010 data for MDRO-related deaths in the US, identifying 154,113 deaths vs an original estimation of 23,000, nearly seven times the original CDC estimate^15^. What we *do* know, is that MDROs are on the rise, with the number of reported MDR strains quadrupling over the last two decades, particularly in young children, accounting for 5–10% hospitalised cases^16–18^.

With the world population now around 8 billion and over 55% of all people concentrated in densely populated urban centres^19^; the risk of a bacterial pandemic is increasingly likely without effective control^20^. The COVID-19 pandemic has been a stark reminder of the ferocity at which an infectious disease can spread and the extensive damage it can cause^21^. Surveillance of infectious agents must improve to allow us to better prepare for and limit future outbreaks, reducing our dependency on antibiotics. Furthermore, tracking how, where, and at what rate antibiotic resistance is evolving in bacteria, can aid in predicting and fighting outbreaks of AMR infections. Currently, patients with suspected infections are most likely to be treated empirically, with some countries estimating 30–50% inappropriate or unnecessary antibiotic usage^22^. This is disappointing considering the significant progress that has been made towards fast, accurate, and affordable diagnostics and the availability of antimicrobial resistance screening.

## Methods used in AMR tracking

The European Committee on Antimicrobial Susceptibility Testing (EUCAST) and the Clinical Laboratory Standards Institute (CLSI) recommend investigating bacterial resistance to antibiotics using culture-based techniques^23–25^: the current gold standard for verifying AMR. Culture-based assessment involves observing and reporting the growth (or absence of growth) of bacteria exposed to various concentrations of antibiotics (Table 1). Culture-based approaches can be used to establish a minimum inhibitory concentration (MIC) or minimum bactericidal concentration (MBC) for a particular organism-antibiotic combination, giving an indication of the likelihood that a particular agent will be clinically effective. The main advantage of assessing AMR this way is the low cost, as the consumables and equipment are inexpensive compared to PCR^26,27^. However, some scientists argue that this labour intensive and slow approach is too costly both in terms of laboratory staff costs and extended in-patient times^26^. Less labour-intensive culture methods do exist, such as Disk and Strip diffusion gradient, but these are still time-costly and laborious to perform when testing multiple samples and antibiotics.

**Table 1:** Description of cultured based antibiotic resistance assessment

Method	Growth media	Description	Resistance indicator
Serial dilution and broth microdilution	Broth or Agar	Antimicrobial agent is serially diluted and added to either broth or agar. Bacteria are then subsequently grown in the media.	Presence of growth indicates resistance.
Disk diffusion	Agar	Bacteria are streaked evenly across plate, paper disks containing antibiotic at known concentrations are added.	Presence of growth around disk indicates resistance. If susceptible a zone of inhibition will be present around disk.
Strip diffusion (gradient method)	Agar	Bacteria are streaked evenly across a plate, and a strip containing an antibiotic gradient concentration is added. The top of the strip been at a much higher concertation to that of the bottom (or vice versa)	The strip has markers indicating the different antibiotic concentrations. The marker at which a zone of inhibition begins is the highest resistance the bacteria can grow up to.

Culture-based assessment relies on the ability to isolate the strain of interest from a complex mixture, and it is also essential that the species is compatible with the culturing technique (e.g. anaerobic bacteria cannot grow in normal atmospheric conditions). To identify strains, a sample is initially grown on solid media and any colonies that form can be identified using a variety of techniques; amplification and sequencing based (16 S and PCR), biochemically (Analytical profile index, API), immunologically (Enzyme-linked immunosorbent assay, ELISA), or through protein fragment analysis using matrix-assisted laser desorption/ionisation time-of-flight mass spectrophotometry (MALDI-TOF MS)^28^. Once the species is/are identified, the antimicrobial susceptibility can be determined. Complex and non-sterile sample types such as faeces make the culture-based assessment difficult, as a plethora of colonies will grow during the initial culturing step. Selective media can be used in this case to target recovery of a suspected microbe of concern^29^.

Lateral flow tests (LFTs) can be used in the context of AMR assessment, the technology uses an immunochromatographic strip impregnated with antibodies to detect key enzymes associated with antimicrobial resistance e.g. beta-lactamase. LFTs proved highly successful during the COVID-19 pandemic^30,31^. However, LFTs have limited use in the context of AMR testing, as there is currently a requirement to first undertake pre-culture step^24^. So, while quick, LFTs are still limited by bacterial growth times and the capacity to undertake these steps^32^.

Molecular techniques for pathogen detection and antibiotic resistance mechanisms are an attractive alternative to culture-based methods due to their high selectivity at the RNA/DNA level, sensitivity, and ability to provide earlier identification (or diagnosis)^24,33,34^. While molecular methods are more expensive than culture-based ones (cost per test), one could argue that the benefit of earlier diagnosis, patient discharge from hospital, and fewer working days lost, presents cost savings in the wider context^26^.

Nucleic acid amplification tests (NAATs) for detection of pathogens can use a variety of amplification methods (PCR, Strand Displacement Amplification (SDA), Transcription-Mediated Amplification), but are mainly limited to PCR for antibiotic resistance gene detection^24^. PCR species identification is highly targeted and requires a level of empirical insight from medical professionals to narrow down the range of causative agents to direct screening. This is also true for AMR – the mechanism of resistance in the pathogen of interest must be known to allow for the design of targeted PCR primers. This is where whole genome sequencing (WGS) presents a huge advantage as it can identify bacteria as well as detect the presence of any AMR genes without prior knowledge^35^, and potentially without the need to culture.

Not all molecular techniques utilize nucleic acids as their form of detection, MALDI-TOF MS investigates the molecular composition of proteins and peptides within a sample. It identifies specific biomarkers based on their mass-to-charge ratio, providing information about the samples molecular profile^36^. This information used in conjunction with a reference database can be used to determine the identity of a pathogen and its AMR profile^37^. MALDI-TOF provides a comprehensive result with the potential to highlight multiple resistance mechanisms, however, this style of analysis can miss certain types of resistance that are not directly related to protein expression, such as mutations in regulatory regions or modifications in non-proteinaceous components of bacteria.

## Sequencing and AMR prediction

Third generation WGS systems provide long reads at high speed, examples being Illumina MiniSeq & MiSeq, and Oxford Nanopore’s MiniON and PromethION^24,38^. These systems permit rapid pathogen identification *and* antibiotic resistance in a single assay without a culturing step. There is ever growing support in the AMR surveillance field that these WGS methods could replace current phenotypic assays^35,39–41^.

To identify which AMR genes are present post-sequencing requires two bioinformatic components: an aligner (e.g. Resistance Gene Identifier (RGI)^39^, AMRFinderPlus^42^ and ResFinder^43^) and a database of known AMR gene sequences and their associated resistance phenotype (e.g. Comprehensive Antibiotic Resistance Database (CARD)^39^, MegaRes^44^ and National Database of Antibiotic Resistant Organisms (NDARO)^45^). WGS analysis provides speed, flexibility, and breadth as clinical samples can be screened against hundreds of potential resistance profiles simultaneously – a process that would be too laborious and excessively time consuming for culture-based approaches. Furthermore, sequencing is neither dependent on pure cultures nor on being able to culture fastidious strains. WGS has great potential for AMR surveillance and diagnosis, but it is not a routine clinical application. Unlike direct phenotypic testing, sequencing predictions only indicate the presence (or absence) of antibiotic resistance sequences in the sample. Clinical and phenotypic information is usually required in order to properly interpret the outputs of sequencing. The presence of an AMR gene does not necessarily translate to antibiotic resistance since the genes may be inactive, an area where MALDI-TOF provides greater certainty of an active resistance owing to its detection of proteins that could be linked to a resistance genes expression^37^. Of greater concern is the observation that, the absence of any AMR indicator genes may not always correctly infer phenotypic susceptibility, a documented example of this can be found in the false negative predictions by WGS in Salmonella enterica^46^.

In 2017, EUCAST highlighted several issues that need to be addressed before the technology can move forward in the clinical context^47^. The key points were:

- There is a lack of evidence for the AMR gene prediction accuracy for many bacteria.
- It is a non-trivial process to establish the equivalent of clinical breakpoints in genomic predictions.
- No standardisation of bioinformatics tools and approach to perform quality control (QC).
- There is no single database of all known resistance genes/mutations - multiple databases developed independently means again that there is no harmonisation and data output is not equivalent.

Nevertheless, the cost of sequencing is coming down and the move towards high throughput methodologies is progressing, meaning that the current barriers to entry are reducing^48^. We are already seeing a strong push towards WGS/NGS sequencing in other diagnostic fields (e.g. genetic disorders) and the value it would add to AMR surveillance and evidence-based drug prescription is significant^49,50^. The creation of standards, both written and physical reference reagents, in this growing field would help to address many of the concerns of EUCAST and help to accelerate wider acceptance.

## Real world examples of early AMR detection potential

To discuss how the different tools (Table 2) perform, we have chosen to highlight real world examples of where screening for antimicrobial resistance is essential or a growing concern:

**Table 2:** Methods utilised in the characterisation of AMR

Method	Summary	Resistance Indicator
Culture	Screen bacteria growth against media containing different concentrations of the antibiotic of interest.	Determination of minimum inhibitory concentration (MIC), through presence or absence of growth.
Matrix-assisted laser desorption/ionisation time-of-flight (MALDI-TOF)	Identifies specific biomarkers associated with resistance mechanisms, through analyses of the mass-to-charge ratio of bacterial proteins or peptides compared to reference databases.	Protein fragment analysis.
Lateral flow test (LFT)	Utilizes antibodies designed to detect enzymes associated with AMR, most commonly cell surface proteins.	Binding to antigens associated with AMR.
Nucleic acid amplification tests (NAAT)	Primers are designed to target specific genes associated with AMR. An amplification step (e.g. PCR) is used to generate a detectable signal.	DNA amplification of gene associated with AMR.
Whole Genome Sequencing (WGS)	By sequencing the entire genome of a bacterial isolate, WGS allows for the identification of specific genetic determinants associated with AMR, including resistance genes, mutations, and mobile genetic elements.	DNA sequence detection associated with AMR

### Infection screening in blood and cerebrospinal fluid (CSF) samples

Blood and CSF samples are commonly used to diagnose bacterial infections, as healthy individuals harbour no bacteria from these sample sites. The normally sterile nature of CSF and blood in these sample sites makes them ideal samples for pathogenic strain detection and identification, as there is no bacterial background against which a pathogen needs to be distinguished from. Nevertheless, despite their diagnostic advantages, there often arises an urgent necessity to treat diseases associated with these samples, such as sepsis or numerous neonatal infections, due to their potentially life-threatening nature. Earlier and effective treatment results in better clinical outcomes, especially in younger patients who are at greater risk from bacterial infections^51–53^. It is standard practice for clinicians to begin empiric antibiotic treatment prior to receiving information on bacterial susceptibility.

A clinical example of CSF usage is Bacterial meningitis, a highly lethal disease^54,55^. Initial empirical treatment is often necessary with CSF samples taken prior to enable informed diagnosis. CSF culture is considered the gold standard; however, PCR is increasingly becoming relied upon because of its far greater sensitivity^55^. It is difficult to employ AMR stewardship, when delays in treatment can cause deaths, but we are beginning to see the results of this with third-generation antibiotics (e.g. ceftriaxone) becoming ineffective against *Escherichia coli* meningitis^56^. The high mortality rate associated with these infections means it is essential we try to move towards faster diagnostic tools to provide early, effective treatment based on evidence (reliable clinical laboratory test results). An ONT (Oxford Nanopore Technologies)-based approach to WGS and rapid diagnostics in blood infections is considered very promising with high accuracy and fast turnaround results, with the potential to be applied to CSF and implemented in clinical settings^57^.

### Sexually transmitted infections (STIs)

STIs are a global problem, with the highest burden in low- and middle- income countries (LMICs). When left untreated, STIs can cause complications ranging from problems with fertility and pregnancy to cancer^58^. The most common bacterial STIs are *Chlamydia trachomatis* and *Neisseria gonorrhoeae* (inferred from Public Health England data^59^). *Treponema pallidum* (syphilis)*, Haemophilus ducreyi* (Chancroid), and *Mycoplasma genitalium* infections are also prevalent but occur at a far lower frequency^59^.

The relatively few causative bacterial agents associated with STIs makes targeted NAAT-based diagnostics an effective solution for infection identification. Furthermore, the characterisation of common AMR causing genes found in *C. trachomatis* and *N. gonorrhoeae*, also lend themselves well to NAAT-based AMR detection^24,60–62^ and the preferred laboratory method for these two strains has shifted from culture^63^ to NAAT^64^ increasing sensitivity and specificity, and faster turnaround time^63^. WGS offers an alternative that would be able to strain ID and screen for AMR at the same time (and rapidly), but the high incidence would be too expensive in comparison to NAAT. However, given the rise in novel AMR causing genes it may become necessary in the future. Indeed, “super” gonorrhoea is already a growing AMR concern, with the first case of drug resistant gonorrhoea reported in London in December 2021^65^ and a further two cases in the UK as of the 7^th^ February 2022 and increasing numbers across Europe^66^. The World Health Organisation (WHO) has launched a global action plan to control the spread and impact of antimicrobial resistance in *N. gonorrhoeae* as part of a wider STI surveillance plan, with a focus on controlling antibiotic usage and disease spread.

### Urinary tract infections (UTIs)

UTI infections are the most common infectious disease after respiratory tract infections and are a major public health problem in terms of morbidity and financial cost^67^. There has been an alarming rise in UTI antimicrobial resistance, likely owing to UTI patients being among the top receivers of outpatient antibiotic prescriptions^67,68^. The leading cause of UTI’s is uropathogenic *Escherichia coli* (UPEC), making up 80% of infections in women aged 18–39^69,70^. The current leading approach to identification and antibiotic susceptibility is culture-based screening. Given the overwhelming amount of UPEC caused infections and the small bacterial background of the sample one could argue for the use of lateral flow or multiplex PCR to confirm presence of *E. coli* and its resistance profile. Although neither of these approaches would rule out other organisms, they provide a far more rapid diagnosis of the leading cause. There is a growth of emerging technologies in UTI diagnostics, utilising microfluidics and lab-on-a-chip concepts to help provide point of care species identification and treatment suggestions^71,72^.

### Upper and lower respiratory tract infections

Respiratory tract infections are a major global health issue, especially in low-income countries with limited healthcare access. Additionally, outbreaks of highly contagious respiratory infections, can have far-reaching consequences on a global scale, causing widespread illness, economic disruption, and loss of life. Efforts to prevent, detect, and effectively manage respiratory tract infections are crucial for safeguarding public health and minimising their impact. Pathogens commonly causing respiratory infections include *Streptococcus pneumoniae*, *Haemophilus influenzae*, *Mycoplasma pneumoniae*, *Pseudomonas aeruginosa*, and *Mycobacterium tuberculosis*^73^. Antibiotics are used to manage these infections with over half of all UK oral antibiotic prescriptions being written for this indication^74^. Several mechanisms conferring antimicrobial resistance in the organisms listed above have been observed and are of increasing concern^73,74^. A recent study describes the life cycle of antibiotic resistance genes in *Pseudomonas aeruginosa* isolated from hospitalised, ventilated patients^75^. They demonstrate the value of using targeted sequencing to identify and track AMR genes, showing that the data generated can inform treatment by enabling patient-specific antibiotic cycling strategies.

Pulmonary tuberculosis (TB) can be transmitted between individuals and is a significant contributor to poor health and mortality rates globally. Prior to the COVID-19 pandemic, TB held the unfortunate distinction of being the leading cause of death among single agent infectious diseases, surpassing even HIV/AIDS in its impact^76^. Culture of sputum of other respiratory secretions is considered the gold standard for diagnosis, however, it is slow-growing, taking two to six weeks for culture and an additional three plus weeks for multi-drug resistance testing^77^. Rapid detection of resistance patterns and prompt initiation of appropriate treatment are essential for effectively controlling TB and minimising the transmission of drug-resistant strains^78^. Faster diagnostic and susceptibility assays already exist (both NAAT and WGS based^79^), with the WHO now pushing for better access to rapid testing^80^. The UK is leading on that front, having implemented the first service for TB rapid diagnostics utilising WGS, shortening TB diagnosis and treatment in a cost-effective way^81^. This successful implementation is a promising first step for a wider adoption of rapid diagnostics in healthcare for other indications.

### Skin and soft tissue infections

Conditions affecting the skin can be both physically painful and disfiguring, leading to both physical discomfort, mental distress and social isolation. Among medical practitioners, dermatologists have the highest prescription rates for antibiotics^82^. Nosocomial (healthcare-associated) infections are a serious complication of severe burns, and the use of systemic prophylactic antibiotics to control infections and reduce sepsis risk has been discussed in several studies over the past several years^83^. These studies have shown that prophylactic use of broad-spectrum antibiotics does not provide protection against sepsis, except for patients with inhalation burns or pneumonia. The overarching theme here is that broad spectrum antibiotics are often used for skin conditions, with little diagnostic or susceptibility screening performed. This needs to change to prevent further AMR evolution. Targeted therapy based on identification of the causative agent and its susceptibility will need to increase in importance.

### Chronic liver disease

Bacterial infections are common in patients with chronic liver disease (CLD) and are one of the most important causes of liver-related complications, progression to liver failure, and mortality in these patients^84^. Resorting to antibiotic prophylaxis and broad-spectrum empirical therapy remains essential in the management of infection prevention in advanced CLD^85^. This approach has however led to the widespread use of antimicrobials, which is the leading cause of the continuous rise of MDRO infections. MDRO rates to quinolone drugs have been recorded up to 40% in CLD patients with spontaneous bacterial peritonitis on prophylactic antibiotics, leading to a break-through recurrence of intra-peritoneal infection. MDR bacteria have emerged as a significant challenge in many countries^86^, and infections caused by these bacteria are associated with a particularly poor prognosis in CLD patients^87^.

Circumventing the harmful impact of AMR in CLD requires a combinatorial approach encompassing antibiotic stewardship programmes, accurate biomarkers of infection onset and resolution, prompting the rapid de-escalation of antimicrobial therapies^88,89^. The other crucial aspect remains the development of rapid testing technologies for the accurate identification of causative pathogens with simultaneous AMR profiling to guide timely and accurate antibacterial therapy in cirrhosis patients^90^ This has paved the way for non-culture-based approaches that offer the potential in reducing the limitations, delays and inaccuracies that are associated with conventional microbiological techniques^91^.

### Microbiome donor screening

Microbiome therapies are a growing area in medicine, offering novel approaches to disease management in instances of unmet clinical need or poor treatment outcomes^92^. Faecal microbiota transplant (FMT) is the first commercially available microbiome treatment, employed to treat recurrent *Clostridioides difficile* infections^93^. In 2019, the first death caused by FMT occurred in the USA when an immunocompromised adult received a FMT that led to an invasive infection caused by extended-spectrum beta-lactamase (ESBL)-producing *E. coli* present in the donor stool^94^. This triggered the FDA to recommend screening for common MDROs^95^ in all donor samples, with the British Society of Gastroenterology and Healthcare Infection Society adopting similar recommendations^96,97^. Even with all these guidelines and safety measures now in place, cases of Shiga toxin *E. coli* infections caused by FMT are still occurring^98,99^, raising concern that pre-treatment screening is not sufficiently robust. Culture based testing for many bacteria may not be consistent or reliable^100,101^. In the case of FMT, false negatives become an unacceptable risk, with the danger of transmitting an infectious organism that was not detected during donor screening.

Molecular methods should be adopted for FMT screening (both for identification of pathogens and AMR assessment) since the sensitivity of this method is much higher than culture. WGS could add further value as it not only identifies strains in a complex mixture and screens for markers of AMR, but it can also provide data that could be used to identify microbiome dysbiosis, potentially before a disease has manifested. Other treatments using microbiome transplants (e.g., vaginal microbiome transplants for bacterial vaginosis) would also benefit from the availability of WGS screening and characterisation (strain ID and AMR).

## Standardisation

The need for standards in antibiotic resistance gene detection by WGS is crucial in combating the global threat of antimicrobial resistance. WGS has emerged as a powerful tool for identifying and characterising antibiotic resistance genes in bacterial genomes. However, the lack of standardised protocols and guidelines for WGS-based resistance profile detection is hindering the accurate and consistent interpretation of results. Biological standardisation is necessary to ensure that different laboratories and researchers are harmonised in quality control measures and data analysis pipelines^102,103^. This will enable reliable comparisons of resistance profiles across studies and facilitate the development of robust surveillance systems. Furthermore, standardised protocols and the use of appropriate reference reagents will promote data sharing and collaboration, allowing for the accumulation of comprehensive and representative datasets that can inform evidence-based policies and interventions. Ultimately, the establishment of written and physical standards in antibiotic resistance gene detection by WGS will enhance our understanding of the global antimicrobial resistance landscape and support efforts to mitigate its impact on public health.

## Conclusion

**Fig. 1:**
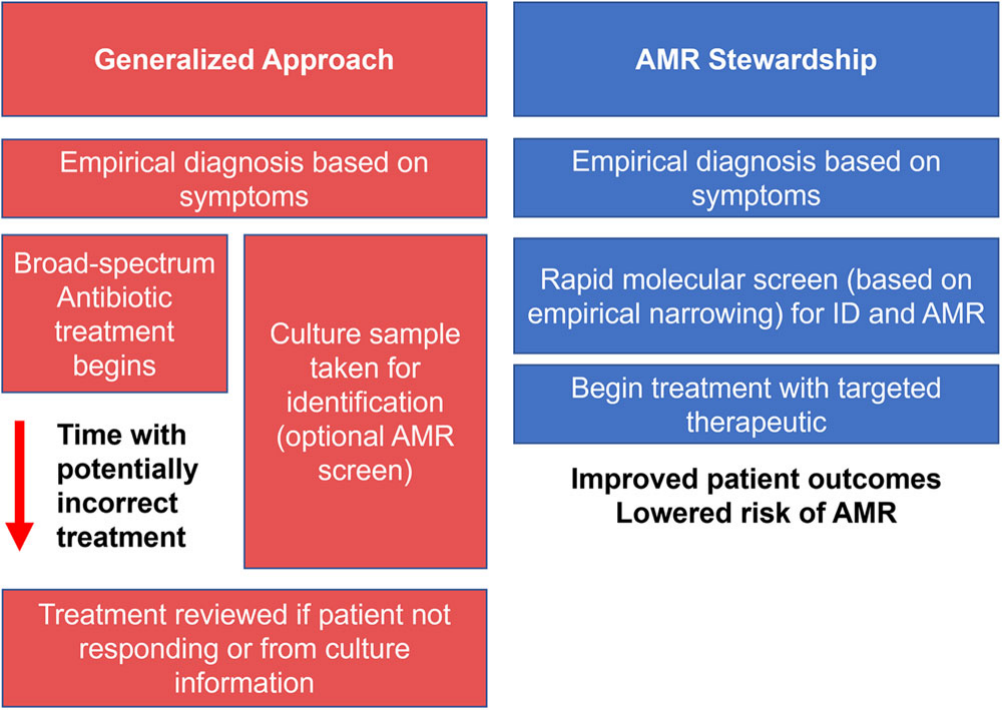
AMR Stewardship through targeted treatments. Current approach for infection diagnostics vs a WGS approach that supports AMR stewardship.

The rise of multi-drug resistant organisms (MDROs) poses a significant threat to global health, leading to increased mortality rates, healthcare and societal costs which necessitate radical intervention. Current methods for AMR detection, most significantly culture-based approaches, have limitations in terms of sensitivity, turnaround time, and the ability to detect all potential resistance genes. WGS offers a promising alternative, providing rapid and comprehensive information about the presence of AMR genes in bacterial strains (Fig. 1). However, several challenges need to be addressed before WGS can be widely implemented in clinical settings. These include the need for standardised methodologies, a comprehensive and unified database of known resistance genes, the availability of appropriate physical reference materials to assure assay performance and the establishment of clinical breakpoints for genomic predictions. Additionally, the cost of sequencing and the interpretation of sequencing results need to be considered to ensure LMICs can also access and derive maximal benefit, as well as optimising upstream processes including biological sample handling and DNA extraction. Despite these challenges, the potential benefits of WGS in AMR surveillance and evidence-based antimicrobial prescription are significant. Establishing standards for WGS-based AMR detection will help address these challenges and accelerate the adoption of this powerful tool in the fight against AMR, ultimately leading to more effective and targeted treatment strategies.
